# Supplementary material for: Genetic engineering of Treponema pallidum subsp. pallidum, the Syphilis Spirochete
Source: PLoS Pathog. 2021 Jul 6;17(7):e1009612. doi: 10.1371/journal.ppat.1009612 (PMC8284648; doi:10.1371/journal.ppat.1009612)
Supplement: S1 Text — (DOC) [file ppat.1009612.s003.doc]

**Insert of the p*tprA*arms-47p-*kan*^R^ vector**

**(pUC57 vector backbone)**

**LEGEND**

In yellow= homology arms (**cloned** into vector)

In green= *tp0574* promoter. In bold are -10, -35 and Ribosomal Binding Site (**cloned i**nto vector)

In gray = kanamycin resistance gene (**cloned** into vector)

Sense Primers are underlined

Antisense primers are double underlined

TATAAAGGCCATTGTGGTTAGTCAGGCAGTTCCCGGCGTTTCAAAGGCGTTTGGGATCATTAAGTCTAAA

CGTCCTGATGTTTTGCTTTTTGCGGGAGAACCACTTGAGCCGGTAGAGATGCTGCAGGAGTCTGCAGACA

TCGTGGTCAGTCAGGACTACTTGTTCGGTGGATATGCCGTTCCGTGGGTTGCGGAAAGGATGGGGGCGCG

CACATTGGTGCATGTCTCTTTTCCCCGGCATATGTCCTACCCCGGTTTGAGGGTTAGGCGTACGGTGATG

AGGGCAGCATGTACCGATTTGGGACTTTCCTTCGCACACGAGGAAGCGCCTGATCCTGTAGACGGTGTCA

GTGACGGAGAACTTGAGGATTTTTTCCACAAGACGATTGTGAAGTGGATCAAAAAATATGGCAAGGAAAC

CCTGTTCTACTGCACCAATGACGCTCACAACAGGCCGCTCATCAGTGCCTTGTTGAAATATGGCGGTATG

CTAATTGGTGCAACCATCTTCGATTACGCTGATGCGCTCGGGGTGCATTATGCTGAGCTTGAAGACGTGT

ATAAAATACGAGAGAAGGTTGAGAAGTCATTGGTTGCCTTCGGCGCAGAGGGGCGCTTTGGATTAAATTT

AAATGCACAGGCATTTACGGTGACCATGGGTTTTGTGGAGTATGCGCGCAAAATCATAGATGGCGAACCG

CGTAAAGATGATATGCGTGAAGCTCTTGCCGAATCCTTCGACTTGTTTACGCGTGACGCACATTGGCGTA

TTGCTCCTTACCTAAGACTGAAAACGCACGAAATTGTTCCGAATCACGTGCTGGTGTATACGGACACATA

CGTCCTGGGTAAATTTACCTTGCCCGTCACAGACCAAGTACTCCCAGAAGGGTATTGGGCATTGACCGCT

AAGGAATAAGAACTCCGTTCGGGTTTTCTGTTTGTAGCCGGGGAGATGGATCGCTTTCTCTGTTTGGCAA

TGTCGCCGTCTCCCTGGGAGCGGATCCTCCCAAAAAGAGGAAGGACGCGCCTGTGTGTGCTCTGCATAAG

ACG**TTGACA**ATCCCTGTGGGGCGT**GCCTATACT**CAGGCCCTCTATAC**GGAG**GTGTAATC ATGAGCCATATTCAACGGGAGACGTCTTGCTCGAGGCCGCGATTAAATTCCAACCTGGATGCTGATTTAT

ATGGGTATAGATGGGCTCGCGATAATGTCGGGCAATCAGGTGCGACAATCTATCGATTGTATGGGAAGCC

CGATGCGCCAGAGTTGTTTCTGAAACATGGCAAAGGTAGCGTTGCCAATGATGTTACAGATGAGATGGTC

AGACTAAACTGGCTGACGGCATTTATGCCTCTTCCGACCATCAAGCATTTTATCCGTACTCCTGATGATG

CATGGTTACTCACCACTGCGATCCCCGGGAAAACAGCATTCCAGGTATTAGAAGAATATCCTGATTCAGG

TGAAAATATTGTTGATGCGCTGGCAGCGTTCCTGCGCCGGTTGCATTCGATTCCTGTTTGTAATTGTCCT

TTTAACAGCGATCGCGTATTTCGTCTCACTCAGGCGCAATCACGAATGAATAACGGTTTGGTTGATGCGA

GTGATTTTGATGACGAGCGTAATGGCTGGCCTGTTGAACAAGTCTGGAAAGAAATGCATAAGCTTTTGCC

ATTCTCACCGGATTCAGTCGTCACTCATGGTGATTTCTCACTTGATAACCTTATTTTTGACGAGGGGAAA

TTAATAGGTTGTATTGATGTTGGACGAGTCGGAATCGCAGACCGATACCAGGATCTTGCCATCCTATGGA

ACTGCCTCGGTGAATTTTCACCTTCATTACAGAAACGGTTTTTTTATAAATATGGCATTGATAATCCTGA

TATGAATAAATTGCAGTTTCATTTGATGCTCGATGAGTTTTTCTGA

AGTTTAGTACAACGATGTCATGTGTCAGATCTAGCAGTATCTGTAATGTATGTTGGTGTACATTAGATAT

TCGTGGGTGGGAAGAAGAGTCACTTTCTGGGGAGGCGTATAGAAGGAACGGGGCGTGGTGTTGTGCCATT

TGTGCGAAAACTGAGTGAAGTAGTGAAAAAAATTACCGCTGACGGGAAAAAATGTTGATCGTGTTTATGA

AAGGGTCATAATGGCTGCCCTATGGGCGCCTGTATATCCGTATATGCGCGTTTTGCGTTAGGGTGTGGGG

TGTTTTTCCTTCATGGTGCGGTTTTGGACGGGGTTTCACGCGCCTTTTCGTCCTCCGCCGCGTTCAGCGG

TTCTGCTGAACTTAGCTGGGGTGTCGTCTTTGATGCAGAAGGTGCCTCTCCAGTTACAGCGGGTAAAAGC

ATACGACATGGGTTTCGCACGAAGAGCAGCTGGAAGCTTGCTTTTCCCTTGTTGCCCAAGAAAGGCGCCA

CGTATACGAGCTTTTCAGGTGAGGATCCCATATGGGTTGAGCTTTCTCTCAAGGGATTGAAGGTGGATTT

TGAAAGTGCTTTAGGGTCGGGAACTGCGGATCCAAGTATGACGACGCGTTCTCCTTTCTTAAAGTCAGGA

AGAAGCGATTTTTCCCTTGAGGCCACACTCCACCTCTACGATGTCTCTTTTTCTGTAGGAAAAGATCCCG

TTTTTCCCTCTAATTTTGCGCAGTTGTGGACCCCCTTTATTACTACTAGTTATGAGTCAAGGAGCGTCAA

ATACGCTCCAGGGTTTGGTGGGGTTGGCGGAAAAATCGCATATCAGGCACGGAATATTTCGAACAGTGGC

ATTACATTCAACTGTGCCCTTTCCTTTTCGTCGAACGGTATATGGAAAAGTGCTCCTTCTGTCACCTCTA

AGGTGAAAGGAAAGGGCACCAATAGTCGGCGCATGCCAGCGGACCCGCACAGTAAATATGGCCTTGGTAC

TGAGTTCACGCTCGTATAC

Green zone primers target the *tp47* promoter.

SENSE AGCGGATCCTCCCAAAAAGA

ANTISENSE GATTACACCTCCGTATAGAG

Yellow zone primers target *tprA* homology arms.

SENSE TGCAACCATCTTCGATTACG

ANTISENSE CGTATGCTTTTACCCGCTGT

Gray zone primers: target the *kan*^R^ gene.

SENSE GAGCCATATTCAACGGGAGA

ANTISENSE ATTCCGACTCGTCCAACATC
